# A comprehensive profile of TCF1^+^ progenitor and TCF1^−^ terminally exhausted PD-1^+^CD8^+^ T cells in head and neck squamous cell carcinoma: implications for prognosis and immunotherapy

**DOI:** 10.1038/s41368-022-00160-w

**Published:** 2022-02-14

**Authors:** Dikan Wang, Juan Fang, Shuqiong Wen, Qunxing Li, Jinming Wang, Lisa Yang, Wenxiao Dai, Huanzi Lu, Junyi Guo, Zhongyan Shan, Wenqiang Xie, Xiangqi Liu, Liling Wen, Jie Shen, Anxun Wang, Qianming Chen, Zhi Wang

**Affiliations:** 1 Hospital of Stomatology, Guanghua School of Stomatology, Guangdong Provincial Key Laboratory of Stomatology, Sun Yat-Sen University, Guangzhou, China; 2 Hospital of Stomatology, Key Laboratory of Oral Biomedical Research of Zhejiang Province, School of Stomatology, Zhejiang University School of Medicine, Hangzhou, China; 3 Department of Oral and Maxillofacial Surgery, First Affiliated Hospital, Sun Yat-Sen University, Guangzhou, China

**Keywords:** Head and neck cancer, Cancer microenvironment

## Abstract

The heterogeneity of exhausted T cells (Tex) is a critical determinant of immune checkpoint blockade therapy efficacy. However, few studies have explored exhausted T cell subpopulations in human cancers. In the present study, we examined samples from two cohorts of 175 patients with head and neck squamous cell cancer (HNSCC) by multiplex immunohistochemistry (mIHC) to investigate two subsets of Tex, CD8^+^PD1^+^TCF1^+^ progenitor exhausted T cells (TCF1^+^Tex^prog^) and CD8^+^PD1^+^TCF1^−^ terminally exhausted T cells (TCF1^−^Tex^term^). Moreover, fresh tumor samples from 34 patients with HNSCC were examined by flow cytometry and immunohistochemistry to further investigate their properties and cytotoxic capabilities and their correlation with regulatory T cells (Tregs) in the tumor immune microenvironment (TIME). mIHC and flow cytometry analysis showed that TCF1^−^Tex^term^ represented a greater proportion of CD8^+^PD1^+^Tex than TCF1^+^Tex^prog^ in most patients. TCF1^+^Tex^prog^ produced abundant TNFα, while TCF1^−^Tex^term^ expressed higher levels of CD103, TIM-3, CTLA-4, and TIGIT. TCF1^−^Tex^term^ exhibited a polyfunctional TNFα^+^GZMB^+^IFNγ^+^ phenotype; and were associated with better overall survival and recurrence-free survival. The results also indicated that larger proportions of TCF1^−^Tex^term^ were accompanied by an increase in the proportion of Tregs. Therefore, it was concluded that TCF1^−^Tex^term^ was the major CD8^+^PD1^+^Tex subset in the HNSCC TIME and that these cells favor patient survival. A high proportion of TCF1^−^Tex^term^ was associated with greater Treg abundance.

## Introduction

Therapeutic advances in immune checkpoint blockade (ICB) therapy have rapidly emerged in the past few years, yielding gratifying results related to the treatment of several kinds of tumors. The programmed death-1 (PD-1)-blocking antibody nivolumab was the first immune checkpoint inhibitor approved by the United States Food and Drug Administration (FDA) for metastatic head and neck squamous cell cancer (HNSCC), receiving approval in 2016 on the basis of the results of the CheckMate 141 trial.^1^ However, due to the complex components of the tumor immune microenvironment (TIME), the overall response rates to ICB therapies for HNSCC range from just 19 to 33%.^1–3^ The HNSCC TIME is populated by dysfunctional immune cells, inhibitory cytokines, and exosomes carrying suppressive ligands.^4,5^ Among all immune cell phenotypes, cytotoxic T cell exhaustion is a hallmark of many cancers and strongly affects ICB therapy.

The term “T cell exhaustion”, which was originally derived from a mouse chronic virus infection model, is now commonly used to define a dysfunctional status of T cells stimulated by continuous antigen burden.^6^ The characteristics of exhausted T cells are a high expression of inhibitory receptors, reduced cytotoxic activity, and reduced proliferation potential.^6,7^ Recently, researchers have made progress in understanding exhausted CD8^+^ T cells (Tex). Two subpopulations of Tex were found in the TIME, progenitor exhausted T cells (Tex^prog^) and terminally exhausted T cells (Tex^term^), and these subpopulations are characterized by distinct epigenetic and transcriptional states.^8–11^ They are distinguished by the expression of TCF1, which is encoded by *TCF7* and plays a fundamental role in T cell development, stemness, and memory formation.^12–14^ TCF1 was highly expressed in Tex^prog^ cells, but not in Tex^term^, thus TCF1 expression could unambiguously identify Tex^prog^ cells.^13^ CD8^+^PD1^+^TCF1^+^ Tex^prog^ (TCF1^+^Tex^prog^) possessed stem-like properties and could be differentiated into Tex^term^ but exhibited relatively lower cytotoxicity. CD8^+^PD1^+^TCF1^−^ Tex^term^ (TCF1^−^Tex^term^) expressed more cytokines and exerted strong antitumor activity. In particular, TCF1^+^ Tex^prog^ were discovered within the tumor region and lymph node and responded preferentially to checkpoint blockade in melanoma.^8,10,15,16^

Previous studies on HNSCC focused mostly on whole tumor-infiltrating CD8^+^ T cells and suggested that the abundance of infiltrating CD8^+^ T cells was associated with better overall survival (OS).^17–19^ Further, a subset of CD8^+^CD103^+^ tissue-resident memory T cells (T_RM_) are critical for protective immunity in HNSCC.^20,21^ However, previous studies have shown that the prognostic value of Tex is controversial. In hepatocellular carcinoma, PD1^hi^CD8^+^ Tex was associated with poor outcomes, whereas in triple-negative breast cancer, they were associated with a better prognosis.^22–25^ A possible explanation might be that the TIME consists of distinct Tex subsets that exhibit distinct cytokine production and phenotypic characteristics. Therefore, to further understand the heterogeneity and prognostic value of PD1^+^CD8^+^Tex, we aimed to explore the profiles of TCF1^+^Tex^prog^ and TCF1^−^Tex^term^ in HNSCC.

In this study, we applied multiplex immunohistochemistry (mIHC) and flow cytometry to investigate the comprehensive profile of TCF1^+^Tex^prog^ and TCF1^−^Tex^term^ in HNSCC, including their spatial distribution, prognostic value, cytokine secretion, and correlation with regulatory T cells (Tregs) in the TIME. We found that CD8^+^PD1^+^TCF1^+^ Tex^prog^ and CD8^+^PD1^+^TCF1^−^ Tex^term^ coexisted in the HNSCC TIME and that the latter was the major subset. The density of tumor-infiltrating TCF1^−^Tex^term^ could serve as an independent prognostic indicator in HNSCC. In addition, the level of infiltrating Tregs increased as the proportion of TCF1^−^Tex^term^ became higher. We propose that these findings could provide new strategies and indications for optimal immunotherapeutic regimens for HNSCC patients.

## Results

### TCF1^−^Tex^term^ was the major PD1^+^CD8^+^Tex subset in the HNSCC TIME

**Fig. 1:**
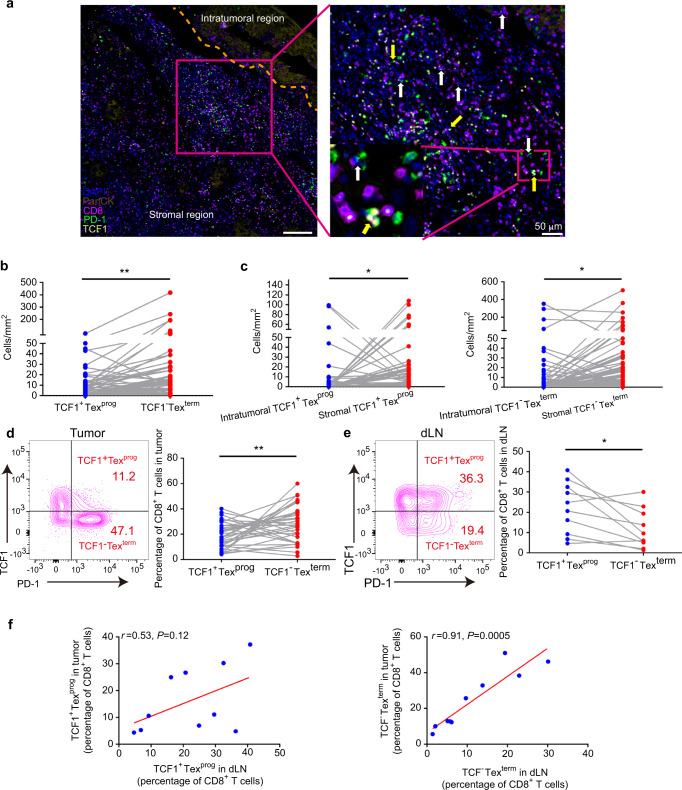
CD8^+^PD-1^+^TCF1^+^ progenitor exhausted T cells (TCF1^+^Tex^prog^) and CD8^+^PD-1^+^TCF1^−^ terminally exhausted T cells (TCF1^−^Tex^term^) in the head and neck squamous cell carcinoma (HNSCC) microenvironment. **a** Representative images of HNSCC multiplex immunohistochemistry (mIHC) slides showing CD8^+^PD-1^+^TCF1^+^ Tex^prog^ (yellow arrow) and CD8^+^PD-1^+^TCF1^−^Tex^term^ (white arrow) cells. **b** Quantitative analysis revealed that the density of TCF1^−^Tex^term^ cells was higher than that of TCF1^+^Tex^prog^ in the whole tumoral region (*n* = 102). **c** Quantitative analysis revealed that progenitor and terminally exhausted T cells were more abundant in the stromal region (*n* = 102). **d** Representative contour plots of TCF1^+^Tex^prog^ and TCF1^−^Tex^term^ in tumor by flow cytometry and quantitative analysis showed that TCF1^−^Tex^term^ were more abundant than TCF1^+^Tex^prog^ in tumors (*n* = 34). **e** Representative contour plots of TCF1^+^Tex^prog^ and TCF1^−^Tex^term^ in draining lymph nodes (dLNs) by flow cytometry and quantitative analysis showed that TCF1^+^Tex^prog^ were more abundant than TCF1^−^Tex^term^ in dLNs (*n* = 10). **f** Flow cytometry results showed that TCF1^−^Tex^term^ in dLNs was positively associated with TCF1^−^Tex^term^ in tumors, while there was no correlation for TCF1^+^Tex^prog^ (*n* = 10). Paired *t* tests were performed for the data in (**b**–**e**). Spearman’s correlation test for (**f**). *P < 0.05, **P < 0.01. Scale bars: 200 μm for left (**a**) and 50 μm for right (**a**)

A five-color mIHC scheme that included anti-PanCK (brown), anti-CD8 (red), anti-PD-1 (green), anti-TCF1 (yellow), and DAPI (blue) was developed to comprehensively profile the spatial distribution of TCF1^+^Tex^prog^ and TCF1^−^Tex^term^. In this scheme, CD8^+^PD-1^+^TCF1^+^ cells represented TCF1^+^Tex^prog^, and CD8^+^PD-1^+^TCF1^−^ cells were TCF1^−^Tex^term^.^8^ Interestingly, these two subtypes were both observed in formalin-fixed, paraffin-embedded (FFPE) samples from HNSCC patients (Fig. 1a). Then, we quantitated the numbers of these two subtypes by StrataQuest software.^26^ We found that TCF1^−^Tex^term^ were significantly more abundant than TCF1^+^Tex^prog^ in the total tumor region (Fig. 1b). To further analyze the spatial distributions of these subpopulations, we deeply classified the intratumoral and stromal regions by PanCK staining (Fig. S1a and Fig. 1a). The results showed that both TCF1^+^Tex^prog^ and TCF1^−^Tex^term^ were significantly enriched in the stroma rather than the intratumoral region (Fig. 1c). In addition, the number of stromal TCF1^−^Tex^term^ was significantly higher than that of stromal TCF1^+^Tex^prog^, but there was no statistical significance in the intratumoral region (Fig. S1b, c).

We further performed flow cytometry to investigate these two types of PD1^+^CD8^+^Tex in 34 fresh HNSCC tumor samples and 10 paired draining lymph nodes (dLNs). The clinicopathologic characteristics of HNSCC patients are shown in Table S1. The flow cytometry gating strategy is displayed in Fig. S1d. We found significantly more CD8^+^PD-1^+^TCF1^−^ Tex^term^ than CD8^+^PD-1^+^TCF1^+^ Tex^prog^ in HNSCC tumor samples which were consistent with the mIHC results (Fig. 1d), whereas, in dLN, the proportion of TCF1^+^Tex^prog^ was significantly higher than that of TCF1^−^Tex^term^ (Fig. 1e). We tried to explore the correlation of Tex subsets between the tumor and dLN. The results showed that TCF1^−^Tex^term^ in dLNs was positively associated with TCF1^−^Tex^term^ in tumors, while there was no correlation for TCF1^+^Tex^prog^ (Fig. 1f). All these results indicated that TCF1^−^Tex^term^ was the major Tex subpopulation in the HNSCC TIME and the proportion of TCF1^−^Tex^term^ in TIME was correlated with that in dLN.

### TCF1^-^Tex^term^ conferred better prognosis in HNSCC

**Fig. 2:**
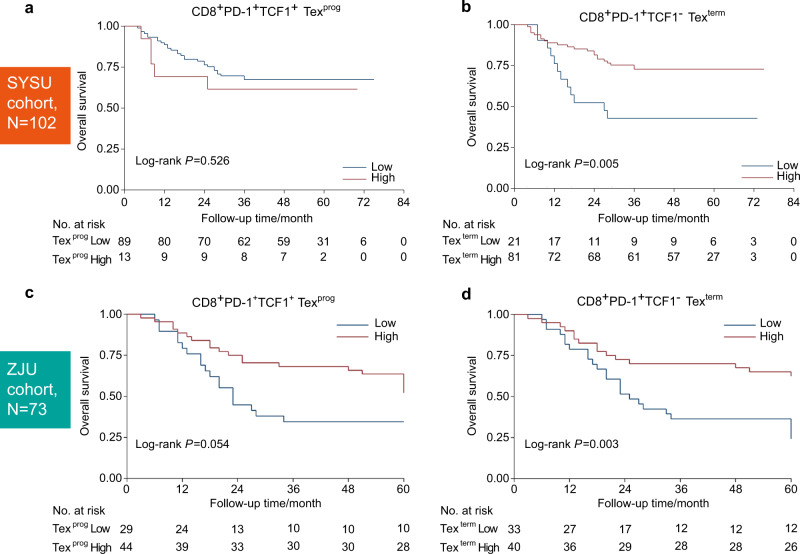
The density of CD8^+^PD-1^+^TCF1^−^ terminally exhausted T cells (TCF1^−^Tex^term^) indicated a favorable prognosis in head and neck squamous cell carcinoma (HNSCC) based on the density from mIHC results. **a** There was no significant overall survival (OS) analysis according to high and low densities of CD8^+^PD-1^+^TCF1^+^ progenitor exhausted T cells (TCF1^+^Tex^prog^) in the SYSU cohort. **b** High density of TCF1^−^Tex^term^ predicted a better OS in the SYSU cohort. **c** There was no significant OS analysis according to high and low densities of TCF1^+^Tex^prog^ in the ZJU cohort. **d** High density of TCF1^−^Tex^term^ indicated a better OS in the ZJU cohort. The log-rank test was used for (**a**–**d**)

As CD8^+^PD-1^+^TCF1^+^ Tex^prog^ and CD8^+^PD-1^+^TCF1^−^ Tex^term^ were detected in HNSCC, we investigated their prognostic value in the Sun Yan-Sen University (SYSU) cohort with 102 patients (patient clinicopathological information is presented in Table S2), based on the density of these two subsets in the mIHC results. We found that the abundance of TCF1^−^Tex^term^ rather than TCF1^+^Tex^prog^ was associated with better OS (Fig. 2a, b) and recurrence-free survival (RFS, Fig. S2a, b). Then, we performed univariate and multivariate OS analyses of multiple clinicopathological parameters in the SYSU cohort. The results showed that among these variables, nodal invasion, clinical-stage, radiotherapy, and TCF1^−^Tex^term^ density were favorable for OS in the univariate log-rank test (Table S3). Then, these variables were entered into multivariate Cox regression analyses. In multivariate analyses, the TCF1^−^Tex^term^ density was an independent prognostic factor for OS (Table 1).

**Table 1:** Multivariate analysis of overall survival based on the Cox’s proportional hazards model in two clinical cohorts

Variables		SYSU cohort		ZJU cohort	
	Items	HR (95% CI)	*P* value	HR (95% CI)	*P* value
Nodal invasion	Negative	Reference		Reference	
	Positive	2.442 (0.977–6.108)	0.056	1.915 (0.779–4.711)	0.157
Clinical stage	Stage I/II	Reference		Reference	
	Stage III/IV	1.054 (0.431–2.577)	0.908	1.389 (0.571–3.378)	0.469
Radiotherapy	Yes	Reference		–	
	No	1.479 (0.694–3.149)	0.311	–	
Tex^term^ density	Low	Reference		Reference	
(cells/mm^2^)	High	0.413 (0.190–0.900)	0.026*	0.352 (0.181–0.676)	0.002*

*Tex*^*prog*^ CD8^+^PD-1^+^TCF1^+^progenitor exhausted T cells, *Tex*^*term*^ CD8^+^PD-1^+^TCF1^−^ terminally exhausted T cells, *SYSU cohort* patients with HNSCC who were treated with surgery at Sun Yat-Sen University, *ZJU cohort* patients with HNSCC who were treated with surgery at Zhejiang University, *HR* hazard ratio, *95% CI* 95% confidence interval, – not included, * *P* < 0.05

To validate the prognostic value of the TCF1^−^Tex^term^ population, we further applied a validation cohort with 73 patients (Zhejiang University cohort, ZJU cohort, patient clinicopathological information is given in Table S2). Consistent with the results from the SYSU cohort, a high density of TCF1^−^Tex^term^ also predicted a better OS and RFS in the ZJU cohort, while there was no statistically significant prognostic difference between patients with high and low densities of TCF1^+^Tex^prog^ (Fig. 2c, d, Fig. S2c, d). Univariate and multivariate OS analyses of multiple clinicopathological parameters in the ZJU cohort also demonstrated that the TCF1^−^Tex^term^ density was indicative of prognosis (Table S3 and Table 1). These results indicated that TCF1^−^Tex^term^ density could serve as an independent prognostic factor for HNSCC.

### TCF1^+^Tex^prog^ and TCF1^−^Tex^term^ exhibited distinct cytokine production and phenotypic characteristics in HNSCC

**Fig. 3:**
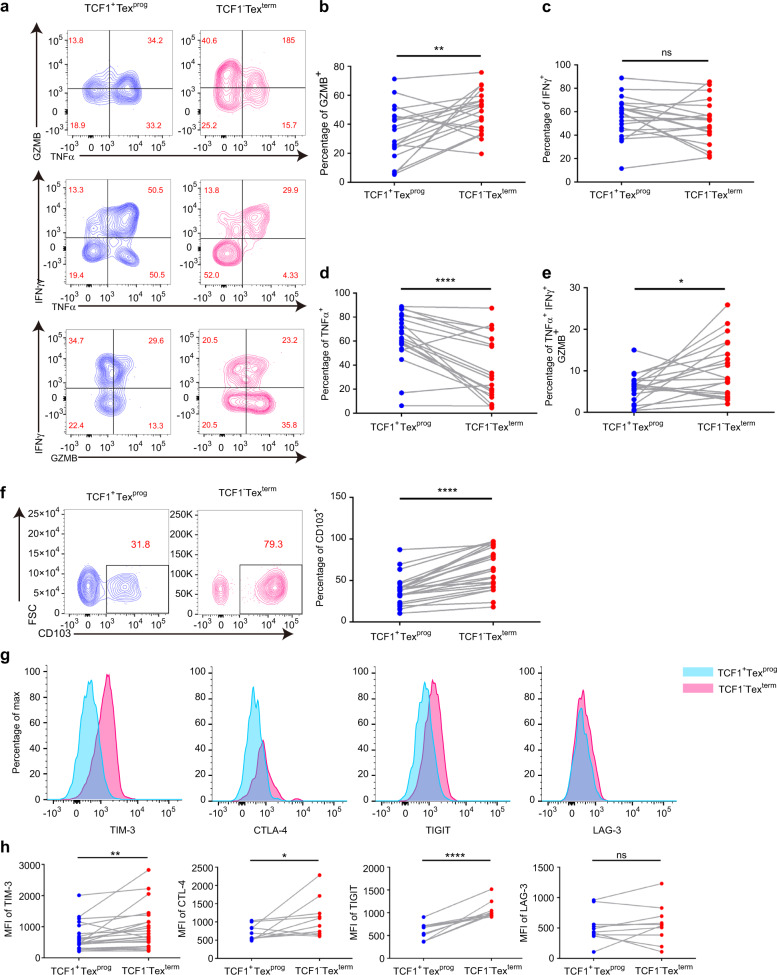
Differences in cytotoxicity activation molecules and cell surface marker expression between CD8^+^PD-1^+^TCF1^+^ progenitor exhausted T cells (TCF1^+^Texprog) and CD8^+^PD-1^+^TCF1^−^ terminally exhausted T cells (TCF1^−^Tex^term^) in head and neck squamous cell carcinoma (HNSCC) tumor tissues were detected using flow cytometry. **a** Representative contour plots of selected cytokine markers (TNFα, GZMB, and IFNγ) gated on TCF1^+^Tex^prog^ or TCF1^−^Tex^term^. **b** TCF1^−^Tex^term^ showed higher GZMB production (*n* = 20). **c** There was no significant difference in IFNγ expression between these two subsets of exhausted T cells (*n* = 20). **d** TCF1^+^Tex^prog^ showed a higher TNFα production (*n* = 20). **e** TCF1^−^Tex^term^ displayed a higher proportion of cells with the polyfunctional phenotype of TNFα^+^GZMB^+^IFNγ^+^ (n = 20). **f** Representative contour plots of CD103 and comparison of CD103 expression among TCF1^+^Tex^prog^ and TCF1^−^Tex^term^ (*n* = 24). **g** Mean fluorescence intensity (MFI) of TIM-3, CTLA-4, TIGIT, and LAG-3 gated on TCF1^+^Tex^prog^ or TCF1^−^Tex^term^. **h** Quantitative analysis of TIM-3 (*n* = 24), CTLA-4 (*n* = 10), TIGIT (*n* = 10), and LAG-3 (*n* = 10) MFI between TCF1^+^Tex^prog^ and TCF1^−^Tex^term^. Paired *t* tests were performed for (**b**–**f** and **h**). *P < 0.05, **P < 0.01, ****P < 0.000 1, ^ns^*P* > 0.05

Based on the different prognostic values of TCF1^+^Tex^prog^ and TCF1^−^Tex^term^, we investigated the cytokine production and phenotypic profiles of these two subsets in fresh tumors. The production capability of interferon-γ (IFNγ), tumor necrosis factor-α (TNFα), and granzyme B (GZMB) was analyzed. TNF-α, IFN-γ, and GZMB are important cytokines that play a critical role in antitumor response. However, there are differences in their functions. TNF-α could promote T cell proliferation and survival and GZMB acts as exocytosis of granule components in the direction of the target cell, leading to a lethal hit of the target cell. We found that GZMB production was significantly higher in CD8^+^PD-1^+^TCF1^−^ Tex^term^ than in CD8^+^PD-1^+^TCF1^+^ Tex^prog^ (Fig. 3a, b). However, there was no significant difference in IFNγ generation between these two subsets (Fig. 3c). In contrast to the case for GZMB, the production of TNFα was significantly higher in TCF1^+^Tex^prog^ than in TCF1^−^Tex^term^ (Fig. 3d). Moreover, TCF1^−^Tex^term^ showed a higher proportion of cells with the TNFα^+^GZMB^+^IFNγ^+^ polyfunctional phenotype (Fig. 3e). It might indicate that highly TNFα-expressing TCF1^+^ Tex^prog^ might associate with its property of proliferation and TCF1^−^ Tex^term^ produced higher GZMB, showing a direct attack on the tumor cells.

In addition, we evaluated the expression of CD103, T cell immunoglobulin and mucin-domain containing-3 (TIM-3), cytotoxic T lymphocyte antigen 4 (CTLA-4), lymphocyte activation gene-3 (LAG-3), and T cell immunoglobulin and ITIM domain (TIGIT) in these two subsets by flow cytometry. CD103 is a marker of T_RM_ that binds to E-cadherin on epithelial cells, and TIM-3, CTLA-4, LAG-3, and TIGIT function as coinhibitors.^5,6^ The flow cytometry results revealed that TCF1^−^Tex^term^ expressed significantly higher levels of CD103 than that of TCF1^+^Tex^prog^ (Fig. 3f). We also found that the mean fluorescence intensity (MFI) of TIM-3, CTLA-4, and TIGIT was stronger in TCF1^−^Tex^term^, but there was no significant difference in the expression of LAG-3 (Fig. 3g, h). These data suggested that TCF1^+^Tex^prog^ produced a higher level of TNFα, and although TCF1^−^Tex^term^ expressed high levels of immune checkpoint receptors, it also expressed higher levels of GZMB, exerting a stronger antitumor response.

### A high proportion of TCF1^−^Tex^term^ was associated with more Treg infiltration

**Fig. 4:**
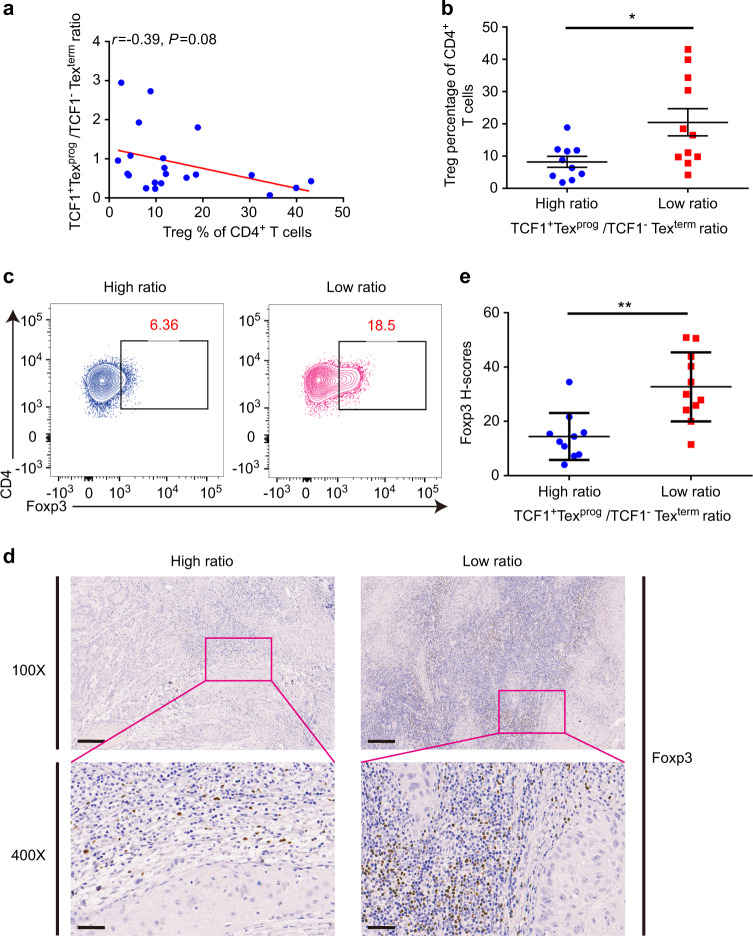
Regulatory T cells (Tregs) increased with a higher proportion of CD8^+^PD-1^+^TCF1^−^ terminally exhausted T cells (TCF1^−^Tex^term^). **a** Tregs negatively correlated with the ratios of progenitor exhausted T cells (TCF1^+^Tex^prog^) to TCF1^−^Tex^term^ (*n* = 21). **b** Flow cytometry result showed that CD4^+^Foxp3^+^Tregs were significantly increased in the low ratio group (high ratio group: *n* = 10, low ratio group: *n* = 11). **c** Representative contour plots of Foxp3^+^Tregs among CD4^+^ T cells in the high/low TCF1^+^Tex^prog^/TCF1^−^Tex^term^ ratio groups. **d**, **e** Representative IHC images of Foxp3 staining in FFPE samples from patients in the high/low ratio groups and quantitative Foxp3 *H*-score analysis showed that Foxp3 expressed higher in the low ratio group. Data are shown as the mean ± SEM. Spearman’s correlation test was performed for a, and unpaired *t* tests were performed for (**b**, **e**). *P < 0.05, **P < 0.01. Scale bars: 200 μm for 100× and 50 μm for 400×

Tregs are increased within tumor sites of various tumor types in humans and mice, limiting the antitumor immune response.^27^ Tregs promote intratumoral T cell exhaustion by modulating the expression of several coinhibitors and the exhaustion-associated transcriptomic signature of CD8^+^ T cells.^28^ We, therefore, used flow cytometry to investigate the association between the percentages of Tregs and the ratios of TCF1^+^Tex^prog^ to TCF1^−^Tex^term^ which represented the status of PD1^+^CD8^+^ Tex proportion. We found that, although not statistically significant, the proportion of CD4^+^Foxp3^+^ Tregs increased as the ratio of TCF1^+^Tex^prog^/TCF1^−^Tex^term^ decreased (Fig. 4a). Then, we further divided these patients into two groups according to the TCF1^+^Tex^prog^/TCF1^−^Tex^term^ ratio in tumors, with the median value as a cutoff. Tumors with a TCF1^+^Tex^prog^/TCF1^−^Tex^term^ ratio ≥ 0.6 were defined as the high ratio group and those with a TCF1^+^Tex^prog^/TCF1^−^Tex^term^ ratio < 0.6 were defined as the low ratio group. We found that the levels of tumor-infiltrating Tregs were significantly higher in the low ratio group than in the high ratio group (Fig. 4b, c). To confirm the flow cytometry results, we further stained Foxp3 in FFPE samples from these patients. In accordance with our findings, Foxp3 was significantly expressed at higher levels in low ratio patients (Fig. 4d, e). Together, these results indicated that a high proportion of TCF1^−^Tex^term^ was associated with more tumor-infiltrating Tregs, which might constrain the persistent antitumor activity of TCF1^−^Tex^term^.

## Discussion

Recent studies have revealed that there are at least two distinct subsets of Tex in melanoma: CD8^+^PD-1^+^TCF1^+^ stem-like Tex^prog^ and CD8^+^PD-1^+^TCF1^−^Tex^term^.^8,10,15^ However, these Tex subsets have not been explored in the context of HNSCC. In this study, we first explored the comprehensive profile of TCF1^+^Tex^prog^ and TCF1^−^Tex^term^ in HNSCC, paving the way to maximize the function of Tex reinvigoration by ICB therapy.

We identified that TCF1^+^Tex^prog^ and TCF1^−^Tex^term^ were both detected in HNSCC by mIHC and flow cytometry. In most HNSCC patients, TCF1^−^Tex^term^ were more abundant than TCF1^+^Tex^prog^ in tumors, consistent with the results in melanoma.^29^ This is partially due to the higher expression of CD103 on TCF1^−^Tex^term^. However, TCF1^+^Tex^prog^ was the main Tex subtype in dLNs. It has been reported that tumor dLNs could act as key orchestrators of antitumor responses induced by ICB therapy and generate the TCF1^+^Tex^prog^ that seed the tumor.^16^ We also found that TCF1^-^Tex^term^ expressed more GZMB and displayed a phenotype of greater polyfunctional TNFα^+^GZMB^+^IFNγ^+^ production, whereas TCF1^+^Tex^prog^ could produce more TNFα which plays an important role in T cell proliferation and survival.^30,31^

Survival analysis revealed that a high abundance of TCF1^−^Tex^term^ rather than TCF1^+^Tex^prog^ predicted better OS and RFS in two clinical cohorts. However, the TCF1^+^Tex^prog^ levels were especially associated with better tumor regression and better overall response in ICB-treated patients.^8,10,32^ Patients with melanoma who had a higher percentage of TCF1^+^Tex^prog^ respond to ICB therapy for a longer duration. Strategies aiming to increase the levels of progenitor and terminally exhausted T cells were proposed by some researchers. Baharom et al. applied a self-assembling nanoparticle vaccine that linked neoantigen peptides to a Toll-like receptor 7/8 agonist (SNP-7/8a) to induce a higher proportion of CD8^+^PD-1^+^TCF1^+^ Tex^prog^, resulting in a superior antitumor response in combination with a PD-L1 blockade.^33^ In addition, Ptpn2 deletion in CD8^+^ T cells enhanced the generation and cytotoxicity of TCF1^−^Tex^term^, promoted tumor control, and improved PD-1 checkpoint blockade responses to B16 tumors.^34^

Even though progenitor and terminally exhausted T cells played important roles in the antitumor response, we noticed that CD4^+^Foxp3^+^ Tregs were elevated as TCF1^+^Tex^prog^/TCF1^−^Tex^term^ ratio decreased. Previous studies reported that high levels of tumor-infiltrating Foxp3^+^ Tregs were associated with better prognosis and locoregional control in HNSCC.^35–37^ However, Foxp3 is a crucial regulator of Tregs that is responsible for a substantial amount of their suppressive potential.^38^ This result indicates that the good prognosticator, Foxp3^+^ Tregs, might be accompanied by high levels of TCF1^-^Tex^term^. Foxp3 expression correlated with decreased TCF1 and reduced accessibility of TCF1-bound chromatin regions.^39^ Treg-derived IL-10 and IL-35 can also directly induce the expression of multiple coinhibitors and drive T cell dysfunction.^28^ This result suggested that Tregs might affect the formation of TCF1^+^Tex^prog^ through transcriptional regulation, resulting in an imbalance in Tex subtypes. In addition, we found that TCF1^−^Tex^term^ expressed higher levels of TIM-3, CTLA-4, and TIGIT, which are markers of a dysfunctional subset of tumor-infiltrating CD8^+^PD1^+^ T cells in cancer. In a transgenic HNSCC mouse model, anti-TIM-3 monoclonal antibody injection induced a reduction in Tregs and increased IFN-γ production on CD8^+^ T cells, suggesting that the antitumor immune response was enhanced by TIM-3 blockade through relieved suppression of negative immune factors.^40^ Therefore, increased Tregs and upregulated inhibitory receptors on TCF1^−^Tex^term^ might construct an immunosuppressive microenvironment in the TIME, resulting in a barrier to the efficacy of immunotherapy. These findings indicated that ICB therapy in combination with Treg deletion might serve as a new strategy to achieve a stronger T cell immune response in the TIME when designing immunotherapy regimens for patients with HNSCC, especially for those with TCF1^+^Tex^prog^/TCF1^−^Tex^term^ ratios lower than 0.6.

In summary, the present study first identified TCF1^−^Tex^term^ as the major subtype in the TIME and then showed that this subtype is indicative of a favorable prognosis in HNSCC. The TCF1^−^Tex^term^ in the tumor environment was correlated with that in dLNs, which showed a higher proportion of Tex^prog^. Moreover, we found that a high proportion of TCF1^−^Tex^term^ was associated with more tumor-infiltrating Tregs. Overall, our study might provide a theoretical basis for optimal ICB immunotherapy in HNSCC.

## Materials and methods

### Patients and study cohorts

A total of 175 consecutive patients’ FFPE specimens with histologically verified primary HNSCC from January 2006 to 2010 came from two clinical centers (Hospital of Stomatology, Guanghua School of Stomatology, Sun Yan-Sen University, SYSU cohort, *N* = 102; The Stomatologic Hospital, School of Medicine, Zhejiang University, ZJU cohort, *N* = 73) and 34 HNSCC patients’ fresh tumor tissues and 10 paired dLNs during surgery between Jan. 2020 and Jun. 2021 were obtained from the Hospital of Stomatology, Guanghua School of Stomatology, Sun Yan-Sen University. All the samples were HPV negative. All the samples used in this study were approved by the ethics committee of the Hospital of Stomatology, Guanghua School of Stomatology, Sun Yat-sen University. Their clinical data were kept anonymous.

### Immunohistochemistry (IHC)

IHC staining was performed on FFPE samples as previously described.^41,42^ Four-micrometer slides were undergone the following procedures: deparaffinized in xylene, rehydrated in graded alcohol, blocked in 3% hydrogen peroxide, antigen retrieval in Tris-EDTA buffer (pH 8.0). Next, the slides were incubated with Foxp3 (#98377S, Cell Signaling Technology, 1:100) primary antibodies at 4 °C overnight. After being treated with HRP goat anti-rabbit secondary antibody, the slides were stained 3,3-diaminobenzidine (DAB). Then Aperio AT2 scanner (Leica) was applied to scan the stained slides. At least three random interested areas (contained both intratumoral and stromal areas) of every section were chosen for quantification. Mean Foxp3 *H*-scores were quantified with background subtraction using Aperio eSlide Manager Quantification software.^43^

### mIHC

Opal 7-color fluorescent IHC kit (NEL811001KT, PerkinElmer) was used to stain the multi-parametric immunofluorescence panel. First, an initial deparaffinization procedure was performed on all slides, followed by fixation in 10% formalin fixation and Tris-EDTA (pH = 9.0) antigen retrieval. Afterward, the slides were incubated with primary antibody, secondary-HRP antibody, and Opal TSA dyes. Subsequently by the other cycles of staining consisted of antigen retrieval, blocking, primary antibody and secondary-HRP antibody incubation, and Opal TSA dye staining. Finally, the slides were mounted with Antifade Reagent (AR1109, BOSTER). DAPI was used for the nuclear counterstain. Images of the whole tissue specimens were acquired by the TissueFAXS platform (TissueGnostics). TCF1^+^Tex^prog^ and TCF1^−^Tex^term^ phenotypes were identified and quantitated using the tissue cytometry of StrataQuest software (V7.0.1.140, TissueGnostics).^44^ Threshold cut-offs depicted by horizontal and/or vertical lines were determined manually by backgating to the greyscale and multiplexed tissue images for each biomarker (CD8, PD-1, TCF1).^45^

The Opal detection fluorophores for exhausted T cells analysis panel was: Opal 690-CD8 (ZA-0508, ZSGB-Biotech, 1:200), Opal 650-PD-1 (#86163S, Cell Signaling Technology, 1:200), Opal 570-TCF1 (#2203S, Cell Signaling Technology, 1:200), Opal 540-PanCK (#4545S, Cell Signaling Technology, 1:500).

### Tissue dissociation

Tissue dissociation was performed as the previous study.^23^ Tumor tissues were cut into several pieces. One-piece was fixed in 10% formalin for IHC or mIHC verification and the other pieces were incubated with 1 mg·mL^−1^ Collagenase IV, 0.5 mg/mL Collagenase I (Gibco) and 0.1 mg·mL^−1^ DNase I (Biofroxx) in complete RPMI 1640 (Gibco) medium with 10% FBS for 30 min at 37 °C. Digested tissues were filtered using a 70 μm cell strainer. Lymph nodes were ground and filtered in the complete medium by a 40 μm cell strainer. Cells were then washed and treated with 1× red blood cell lysis buffer (BD Biosciences) for 2 min at room temperature. After washing with PBS (Gibco), the cell suspensions were then used for flow cytometry analysis.

### Flow cytometry

For intra-nuclear staining, the samples were first stained with Viability Ghost Dye (Tonbo Biosciences) for 15 min at room temperature, then incubated with the cell surface markers: CD45 (2D1, eBioscience), CD20 (2H7, eBioscience), CD3 (OKT3, Tonbo Biosciences), CD4 (OKT4, Biolegend), CD8 (SK1, eBioscience), PD-1 (J505, eBioscience), CD103 (Ber-ACT8, Biolegend), TIM-3 (F38-2E2, eBioscience), TIGIT (VSTM3, Biolegend), LAG-3 (7H2C65, Biolegend) for 30 min at 4 °C. Afterward, Foxp3/Transcription Factor Staining Buffer Set (Tonbo Biosciences) was applied according to the manufacturer’s instructions for the next nuclear molecules staining. TCF1 (#2203S, Cell Signaling Technology, 1:400) was stained for 45 min at room temperature. Lastly, Alexa Fluor 488 donkey anti-rabbit secondary antibody (#2156521, Thermo Fisher Scientific, 1:500), CTLA-4 (14D3, eBioscience), and Foxp3 (PCH101, eBioscience) were stained.

For cytokine detection, the samples were stimulated for 5 h with a cell stimulation cocktail (Tonbo Biosciences) at 37 °C, 5% CO_2_. Then the samples were washed with PBS and stained with Viability Ghost Dye. Next incubated with the membrane molecules: CD3, CD4, CD8, and PD-1. Followed by Foxp3/Transcription Factor Staining Buffer Set application and TCF1 staining. Lastly, the cell suspensions were incubated with donkey anti-rabbit secondary antibody, TNFα (MAB11, eBioscience), GZMB (CB11, eBioscience), IFNγ (4S.B3, Biolegend) overnight. Stained cells were washed and resuspended in PBS. All the samples were acquired on BD LSRFortessa (BD Biosciences). Flow cytometry data were analyzed by FlowJo software (Tree Star).

### Statistical analysis

All results were summarized as mean ± SEM, and statistical analysis was performed with Prism 7.0 (Graphpad Software). Differences between groups were evaluated by paired or unpaired two-tailed Student’s *t* test. To determine the optimal cutoff point for the high- and low-density groups in our cohort and validation cohort, we used the maximally selected rank statistics from the “maxstat” R package.^46,47^ The cutoff point for RFS was set to the same as the value for OS analysis. Survival analysis was performed and presented by using the Kaplan–Meier method. The difference in survival curves was tested by the log-rank test. The correlation analysis between groups was determined by Spearman’s correlation test. *P* < 0.05 was considered statistically significant (*P < 0.05, **P < 0.01, ***P < 0.001, ****P < 0.000 1, ^ns^*P* > 0.05).

## Supplementary information

Supplementary materials

## Data Availability

Data are available upon reasonable request. All data relevant to the study are included in the article or uploaded as supplementary information.
