# Supplementary material for: A comprehensive profile of TCF1+ progenitor and TCF1− terminally exhausted PD-1+CD8+ T cells in head and neck squamous cell carcinoma: implications for prognosis and immunotherapy
Source: Int J Oral Sci. 2022 Feb 14;14:8. doi: 10.1038/s41368-022-00160-w (PMC8841504; doi:10.1038/s41368-022-00160-w)
Supplement: Supplementary file 1 — Supplementary materials [file 41368_2022_160_MOESM1_ESM.docx]

**Supplementary materials**

**A comprehensive profile of TCF1^+^ progenitor and TCF1^-^ terminally exhausted PD-1^+^CD8^+^ T cells in head and neck squamous cell carcinoma: implications for prognosis and immunotherapy**

Dikan Wang^1^, Juan Fang^1^, Shuqiong Wen^1^, Qunxing Li^1^, Jinming Wang^1^, Lisa Yang^1^, Wenxiao Dai^1^, Huanzi Lu^1^, Junyi Guo^1^, Zhongyan Shan^1^, Wenqiang Xie^1^, Xiangqi Liu^1^, Liling Wen^1^, Jie Shen^3^, Anxun Wang^2^, Qianming Chen^3^, Zhi Wang^1^

^1^Hospital of Stomatology, Guanghua School of Stomatology, Guangdong Provincial Key Laboratory of Stomatology, Sun Yat-Sen University, Guangzhou, Guangdong, China.

^2^Department of Oral and Maxillofacial Surgery, First Affiliated Hospital, Sun Yat-Sen University, Guangzhou, Guangdong, China.

^3^Hospital of Stomatology, Key Laboratory of Oral Biomedical Research of Zhejiang Province, School of Stomatology, Zhejiang University School of Medicine, Hangzhou, Zhejiang, China.

Correspondence: Zhi Wang ([wangzh75@mail.sysu.edu.cn](mailto:wangzh75@mail.sysu.edu.cn))

These author contributed equally: Dikan Wang, Juan Fang, Shuqiong Wen

**Fig. S1**

**
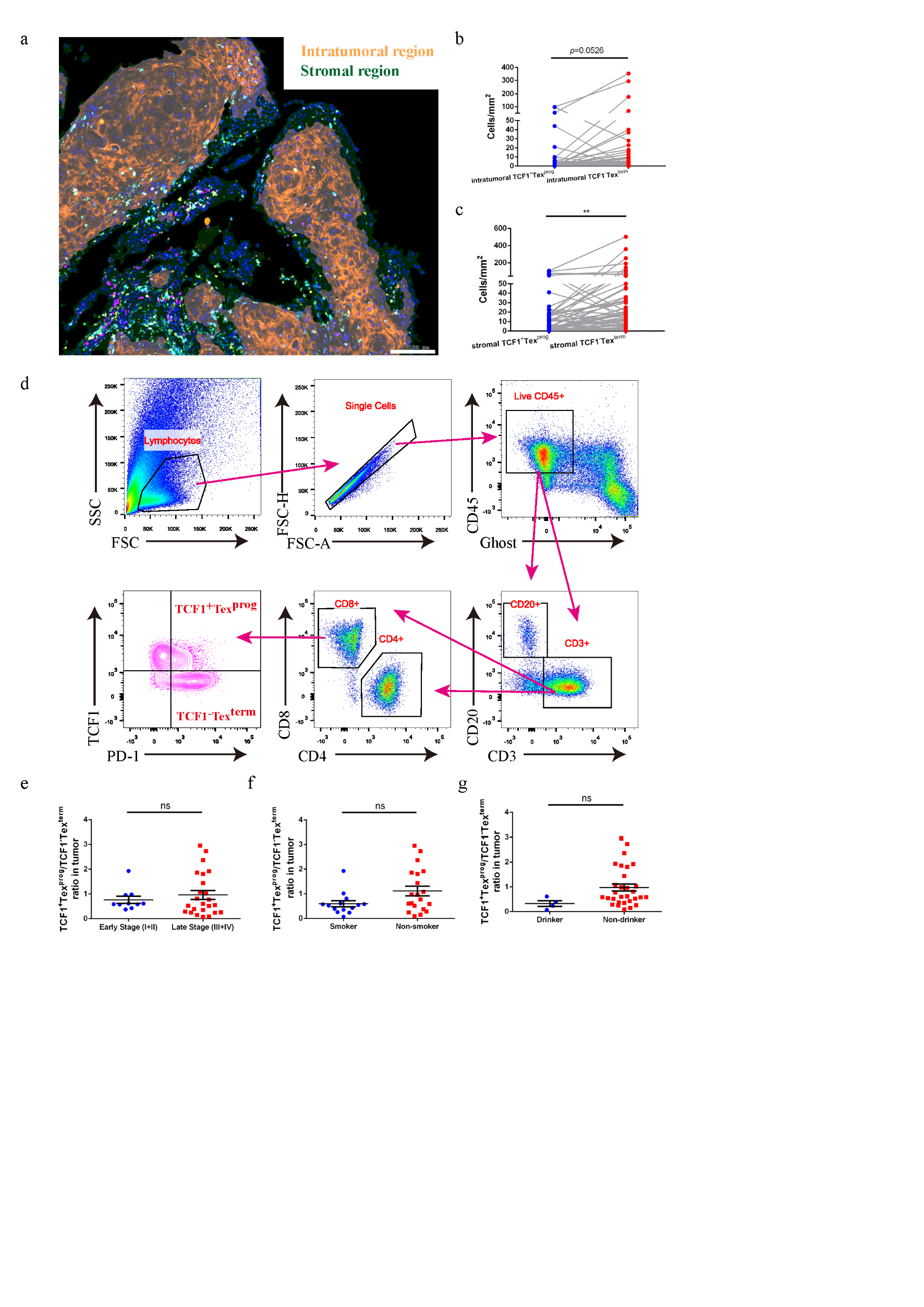
**

**Fig. S1**. **a** Representative mIHC image to show intratumoral and stromal region recognition. **b** and **c** mIHC quantitative analysis revealed that both intratumoral and stromal TCF1^-^Tex^term^ were more than TCF1^+^Tex^prog^, particularly in the stromal region (n=102). **d** Gating strategy to detect and classify CD8^+^PD-1^+^TCF1^+^Tex^prog^ and CD8^+^PD-1^+^TCF1^-^Tex^term^ by flow cytometry. Data were shown as Mean ± SEM. Paired t test for b and c. **: *P* < 0.01, ns: *P* >0.05. Scale bars: 200 μm for a.

**Fig. S2**

**
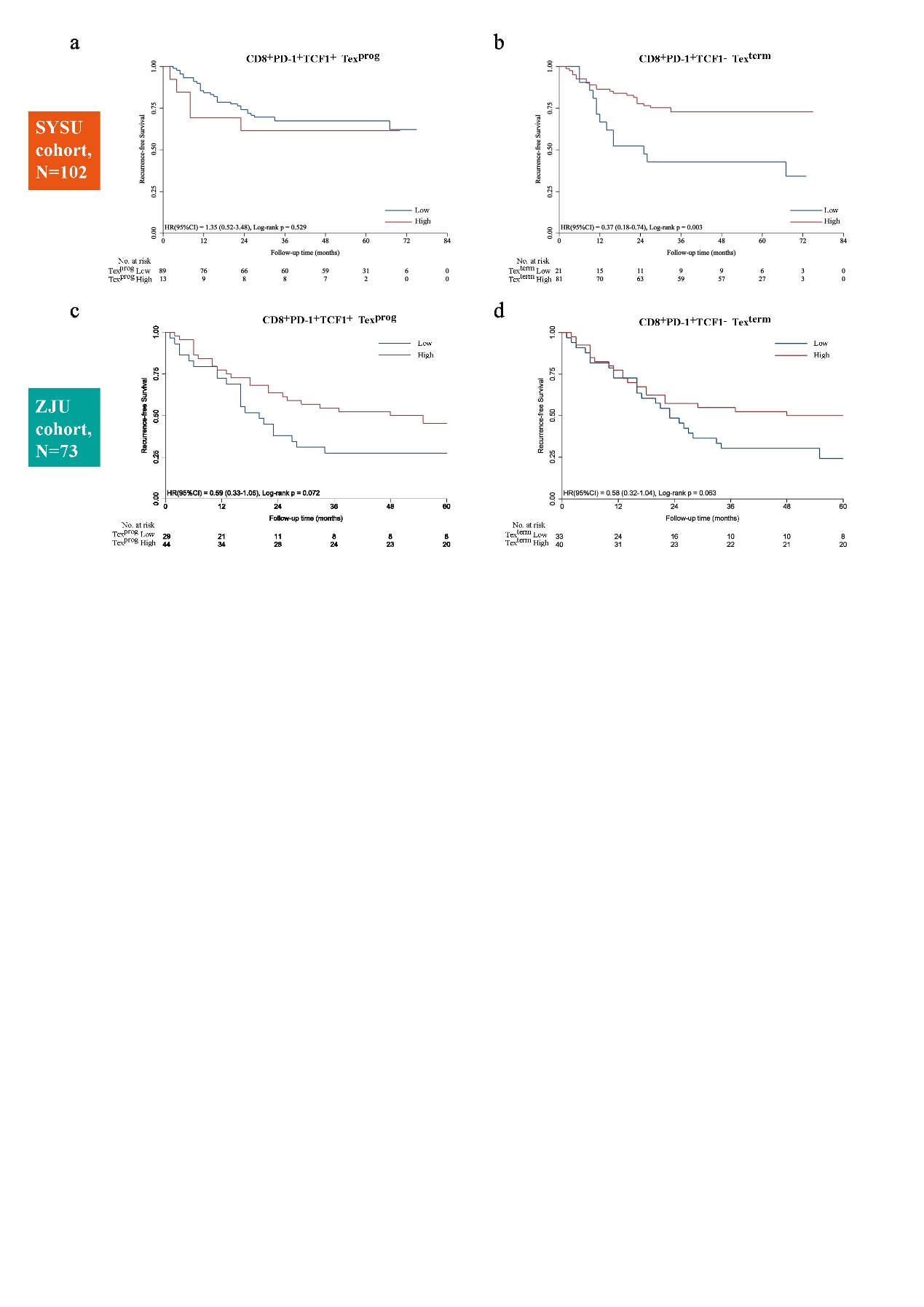
**

**Fig. S2**. CD8^+^PD-1^+^TCF1^-^ terminally exhausted T cells (TCF1^-^Tex^term^) indicated a favorable recurrence-free survival (RFS) in head and neck squamous cell carcinoma (HNSCC). **a** Kaplan-Meier curves for RFS according to high and low density of CD8^+^PD-1^+^TCF1^+^ progenitor exhausted T cells (TCF1^+^Tex^prog^) in the SYSU cohort. **b** Kaplan-Meier curves for RFS according to high and low density of TCF1^-^Tex^term^ in the SYSU cohort. **c** RFS analysis of high/low density of TCF1^+^Tex^prog^ in the ZJU cohort. **d** RFS analysis of high/low density of TCF1^-^Tex^term^ in the ZJU cohort. The log-rank test was used for a-d.

**Table S1.** Clinicopathologic characteristics of the HNSCC patients in the flow cytometry cohort

| Patient# | Gender | Age | Cigarette smoking | Alcohol drinking | Primary site | HPV status | TNM classification | Clinical stage | Differentiation | Neoadjuvant therapy |
| --- | --- | --- | --- | --- | --- | --- | --- | --- | --- | --- |
| 1 | Male | 80 | No | No | Base of tongue | Negative | T2N0M0 | Ⅱ | Moderate | None |
| 2 | Female | 32 | No | No | Tongue | Negative | T2N1M0 | Ⅲ | Moderate | None |
| 3 | Female | 63 | No | No | Tongue | Negative | T1N0M0 | Ⅰ | Moderate | None |
| 4 | Male | 58 | Yes | No | Tongue | Negative | T2N0M0 | Ⅱ | Moderate | None |
| 5 | Female | 57 | No | No | Gingiva | Negative | T2N0M0 | Ⅱ | Well | None |
| 6 | Male | 69 | Yes | Yes | Tongue | Negative | T1N0M0 | Ⅰ | Moderate | None |
| 7 | Female | 75 | No | No | Tongue | Negative | T1N0M0 | Ⅰ | Well | None |
| 8 | Female | 56 | No | No | Buccal | Negative | T3N2bM0 | Ⅳ | Moderate | None |
| 9 | Female | 73 | No | No | Tongue | Negative | T2N1M0 | Ⅲ | Moderate | None |
| 10 | Male | 43 | No | No | Buccal | Negative | T2N2bM0 | Ⅳ | Moderate | None |
| 11 | Male | 38 | Yes | No | Base of tongue | Negative | T2N1M0 | Ⅲ | Well | Radiotherapy |
| 12 | Female | 71 | No | No | Gingiva | Negative | T4aN2bM0 | Ⅳ | Moderate | None |
| 13 | Male | 86 | No | No | Buccal | Negative | T2N0M0 | Ⅱ | Moderate | None |
| 14 | Male | 48 | No | No | Oropharynx | Negative | T4aN2bM0 | Ⅳ | Moderate | None |
| 15 | Male | 46 | Yes | Yes | Base of tongue | Negative | T2N1M0 | Ⅲ | Well | None |
| 16 | Male | 42 | Yes | No | Tongue | Negative | T2N2bM0 | Ⅳ | Poor | None |
| 17 | Male | 48 | No | No | Tongue | Negative | T3N2bM0 | Ⅳ | Well | None |
| 18 | Female | 83 | No | No | Tongue | Negative | T4aN1M0 | Ⅳ | Moderate | Chemotherapy |
| 19 | Male | 49 | Yes | No | Tongue | Negative | T1N0M0 | Ⅰ | Poor | None |
| 20 | Female | 68 | No | No | Tongue | Negative | T2N1M0 | Ⅲ | Well | None |
| 21 | Male | 64 | Yes | Yes | Gingiva | Negative | T4N0M0 | Ⅳ | Moderate | None |
| 22 | Male | 56 | Yes | No | Tongue | Negative | T4aN1M0 | Ⅳ | Moderate | None |
| 23 | Male | 63 | Yes | Yes | Tongue | Negative | T2N0M0 | Ⅱ | Moderate | None |
| 24 | Male | 56 | Yes | No | Tongue | Negative | T2N0M0 | Ⅱ | Poor | None |
| 25 | Male | 65 | No | No | Gingiva | Negative | T4N0M0 | IV | Poor | None |
| 26 | Female | 50 | No | No | Buccal | Negative | T4aN0M0 | IV | Moderate | None |
| 27 | Male | 67 | Yes | No | Tongue | Negative | T2N1M0 | III | Well | None |
| 28 | Female | 62 | No | No | Buccal | Negative | T2N2bM0 | IV | Poor | None |
| 29 | Female | 71 | No | No | Gingiva | Negative | T4aN2bM0 | IV | Moderate | None |
| 30 | Female | 74 | No | No | Floor of mouth | Negative | T3N0M0 | III | Well | None |
| 31 | Male | 66 | Yes | No | Tongue | Negative | T1N1M0 | III | Moderate | None |
| 32 | Male | 59 | Yes | No | Tongue | Negative | T3N0M0 | III | Moderate | None |
| 33 | Male | 50 | Yes | No | Tongue | Negative | T3N2bM0 | IV | Well | None |
| 34 | Male | 63 | No | No | Floor of mouth | Negative | T3N0M0 | III | Well | None |

| Variables | SYSU cohort (N=102) | |  | ZJU cohort (N=73) | |
| --- | --- | --- | --- | --- | --- |
|  | No. | % |  | No. | % |
| Age (years) | 59 (32-85) |  |  | 60 (24-85) |  |
| Gender |  |  |  |  |  |
| Male | 70 | 68.6 |  | 51 | 69.9 |
| Female | 32 | 31.4 |  | 22 | 30.1 |
| Cigarette smoking | 44 | 43.9 |  | 40 | 54.8 |
| Alcohol drinking | 37 | 36.3 |  | 36 | 49.3 |
| Primary site |  |  |  |  |  |
| Tongue | 45 | 44.1 |  | 30 | 41.1 |
| Buccal mucosa | 20 | 19.6 |  | 12 | 16.5 |
| Gingiva | 17 | 16.7 |  | 11 | 15.1 |
| Floor of mouth | 10 | 9.8 |  | 5 | 6.8 |
| Palate | 6 | 5.9 |  | 5 | 6.8 |
| Others | 4 | 3.9 |  | 10 | 13.7 |
| Differentiation |  |  |  |  |  |
| Well | 77 | 75.5 |  | 51 | 69.9 |
| Moderate | 20 | 19.6 |  | 18 | 24.7 |
| Poor | 5 | 4.9 |  | 4 | 5.4 |
| Tumor size |  |  |  |  |  |
| T1 | 24 | 23.5 |  | 20 | 27.4 |
| T2 | 44 | 43.1 |  | 34 | 46.6 |
| T3 | 11 | 10.8 |  | 9 | 12.3 |
| T4 | 23 | 22.5 |  | 10 | 13.7 |
| N stage |  |  |  |  |  |
| N0 | 77 | 75.5 |  | 49 | 67.1 |
| N1-N3 | 25 | 24.5 |  | 24 | 32.9 |
| Clinical stage |  |  |  |  |  |
| Early stage (I +II) | 57 | 55.9 |  | 36 | 49.3 |
| Late stage (III + IV) | 45 | 44.1 |  | 37 | 50.7 |

**Table S2.** Demographic and clinical information of the patients from two clinical cohorts in the survival analysis cohort

**Table S3.** Univariate overall survival analysis of clinicopathological parameters in two clinical cohorts

| Variables |  | SYSU cohort | | ZJU cohort | |
| --- | --- | --- | --- | --- | --- |
|  |  | HR (95% CI) | *P* Value | HR (95% CI) | *P* Value |
| Gender | Male | 0.551(0.240-1.266) | 0.160 | 0.766(0.374-1.568) | 0.466 |
|  | Female |  |  |  |  |
| Age (years) | ≥60 | 1.807(0.918-3.559) | 0.087 | 0.640(0.343-1.192) | 0.159 |
|  | <60 |  |  |  |  |
| Smoking | Yes | 1.937(0.984-3.813) | 0.056 | 1.036(0.553-1.940) | 0.911 |
|  | No |  |  |  |  |
| Alcohol | Yes | 1.339(0.676-2.652) | 0.402 | 1.544(0.824-2.894) | 0.175 |
|  | No |  |  |  |  |
| Primary site | Tongue | 0.801(0.611-1.050) | 0.109 | 1.269(0.778-2.070) | 0.340 |
|  | Buccal |  |  |  |  |
|  | Gingiva |  |  |  |  |
|  | Mouth Floor |  |  |  |  |
|  | Palate |  |  |  |  |
|  | Others |  |  |  |  |
| Differentiation | High | 1.050(0.584-1.888) | 0.871 | 1.015(0.849-1.213) | 0.871 |
|  | Moderate |  |  |  |  |
|  | Poor |  |  |  |  |
| T stage | T1/T2 | 1.230(0.616-2.458) | 0.557 | 1.198(0.598-2.401) | 0.610 |
|  | T3/T4 |  |  |  |  |
| Nodal invasion | Positive | 2.755(1.389-5.463) | 0.004* | 2.102(1.116-3.962) | 0.022* |
|  | Negative |  |  |  |  |
| Clinical stage | Stage I/II | 2.131(1.075-4.222) | 0.030* | 1.971(1.043-3.725) | 0.037* |
|  | Stage III/IV |  |  |  |  |
| Radiotherapy | Yes | 2.314(1.175-4.558) | 0.015* | 0.628(0.151-2.607) | 0.522 |
|  | No |  |  |  |  |
| Chemotherapy | Yes | 1.208(0.526-2.774) | 0.656 | 0.988(0.530-1.842) | 0.969 |
|  | No |  |  |  |  |
| Tex^prog^ density | High | 1.356(0.525-3.504) | 0.530 | 1.004(0.995-1.013) | 0.358 |
| (cells/mm^2^) | Low |  |  |  |  |
| Tex^term^ density | High | 0.378(0.186-0.767) | 0.007* | 0.391(0.205-0.744) | 0.004* |
| (cells/mm^2^) | Low |  |  |  |  |

Tex^prog^: CD8^+^PD1^+^TCF1^+^progenitor exhausted T cells; Tex^term^: CD8^+^PD1^+^TCF1^-^terminally exhausted T cells; SYSU cohort: patients with HNSCC who were treated with surgery at Sun Yat-Sen University; ZJU cohort: patients with HNSCC who were treated with surgery at Zhejiang University; HR: hazard ratio; 95% CI: 95% confidence interval; * *P*<0.05.
